# On Nucleation Pathways and Particle Size Distribution Evolutions in Stratospheric Aircraft Exhaust Plumes with H_2_SO_4_ Enhancement

**DOI:** 10.1021/acs.est.3c08408

**Published:** 2024-04-09

**Authors:** Fangqun Yu, Bruce E. Anderson, Jeffrey R. Pierce, Alex Wong, Arshad Nair, Gan Luo, Jason Herb

**Affiliations:** † Atmospheric Sciences Research Center, State University of New York, Albany, New York 12226, United States; ‡ Science Directorate, NASA Langley Research Center, Hampton, Virginia 23666, United States; § Department of Atmospheric Science, Colorado State University, Fort Collins, Colorado 80521, United States; ∥ SilverLining, 500 North Capitol St NW, #210, Washington, District of Columbia 20001, United States

**Keywords:** solar radiation modification, stratospheric aerosol injection, H_2_SO_4_ injection, particle nucleation, aircraft exhaust plume

## Abstract

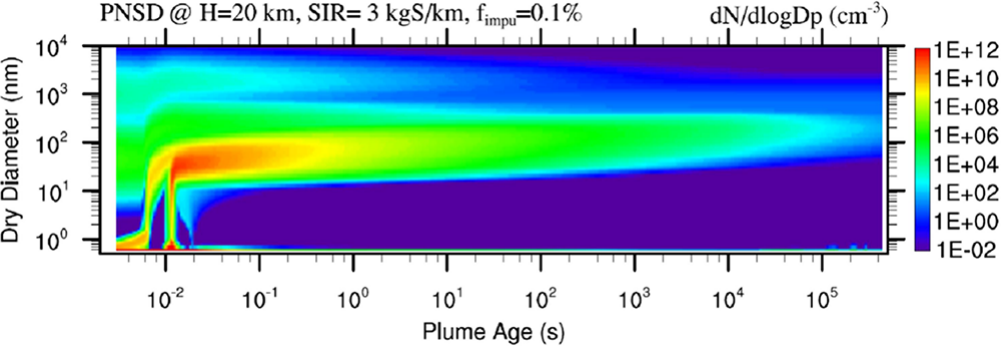

Stratospheric aerosol injection (SAI) is proposed as a means of reducing global warming and climate change impacts. Similar to aerosol enhancements produced by volcanic eruptions, introducing particles into the stratosphere would reflect sunlight and reduce the level of warming. However, uncertainties remain about the roles of nucleation mechanisms, ionized molecules, impurities (unevaporated residuals of injected precursors), and ambient conditions in the generation of SAI particles optimally sized to reflect sunlight. Here, we use a kinetic ion-mediated and homogeneous nucleation model to study the formation of H_2_SO_4_ particles in aircraft exhaust plumes with direct injection of H_2_SO_4_ vapor. We find that under the conditions that produce particles of desired sizes (diameter ∼200–300 nm), nucleation occurs in the nascent (*t* < 0.01 s), hot (*T* = 360–445 K), and dry (RH = 0.01–0.1%) plume and is predominantly unary. Nucleation on chemiions occurs first, followed by neutral new particle formation, which converts most of the injected H_2_SO_4_ vapor to particles. Coagulation in the aging and diluting plumes governs the subsequent evolution to a narrow (σ_g_ = 1.3) particle size distribution. Scavenging by exhaust soot is negligible, but scavenging by acid impurities or incomplete H_2_SO_4_ evaporation in the hot exhaust plume and enhanced background aerosols can matter. This research highlights the need to obtain laboratory and/or real-world experiment data to verify the model prediction.

## Abstract 2

Solar climate interventions through stratospheric aerosol injection have received increasing attention. Here, we address critical knowledge gaps in particle nucleation and subsequent growth in H_2_SO_4_ injection plumes unresolved in global models.

## 1 Introduction

To respond to the ongoing climate crisis, the top priority is to rapidly reduce emissions of carbon dioxide and other greenhouse gases, which are the root drivers of recent and projected global warming.^1^ Nevertheless, because of the challenges of cutting emissions at adequate rates and the long lifetime of greenhouse gases, the necessity to understand the full range of options available for protecting the safety of human and natural systems has been emphasized in a recent report by the US National Academies of Sciences, Engineering and Medicine.^2^ Solar radiation modification/management (SRM) has received increasing attention as a transitionary tool for limiting the global surface temperature increase below 1.5 °C and buying time for carbon emission reduction and removal.^2−6^ The effectiveness and potential risks of stratospheric aerosol injection (SAI) in modifying Earth’s climate have been studied using global models,^7−9^ but little attention has been given to plume processes that are not resolved in global models.

The SAI efficacy has been well recognized to be a function of the sizes of injected aerosols, with the peak efficacy in the 200–300 nm range.^4,10−12^ While coagulation is known to be important in governing the steady-state size distributions of stratospheric aerosols, other processes are likely to be important for SAI efficacy as well which can be seen from the large difference in SAI efficacy for H_2_SO_4_ and SO_2_ injections in several model studies.^8^ In realistic SAI scenarios, the stratospheric aerosols are unlikely to be in a steady state (or equilibrium) because aerosols (or precursors) to be injected continuously are unlikely to be homogeneous both spatially or temporally. In such situations, subgrid plume scale processes are important. Two critical issues limiting our understanding of SRM scenarios are the accurate representation of introducing aerosols or their precursors into the stratosphere and subgrid plume scale process-level understanding to create particles of desired sizes.^4,10,11^ The NASEM report highlighted key questions on this topic, including: “Do ions generated in the engine enhance the rates of nucleation significantly (i.e., by a factor of 10 or more)? Given the results of the items above, are existing models of nucleation, aerosol dynamics, and plume dispersion sufficient to adequately predict the timing and properties of the particle size distribution for a given input of aerosol or precursor over a range of altitudes and latitudes?”.^2^ Not considering these plume-scale processes may result in the misrepresentation of the relationship between the amount of injected sulfur and the computed aerosol size distribution and thus the uncertainty in the calculated SAI efficiency.

Aircraft are a likely platform for SAI and a few existing SAI plume scale studies have explored using this platform to introduce aerosols into the stratosphere.^2,4,10,13^ Rasch et al.^4^ analyzed the evolution of aerosols injected directly into the stratosphere from a jet-fighter-sized aircraft, using the analytical solutions of the aerosol number concentration evolution in an expanding aircraft plume.^14^ They showed that aerosol properties in the aircraft injection plume can be severely affected by self-coagulation and coagulation scavenging by the background aerosol. Rasch et al.^4^ mentioned the potential physical limitations of nucleation processes, including chemiion nucleation,^15^ to injection schemes but did not explicitly calculate nucleation rates. Motivated by a previous analysis^16^ suggesting that the SO_2_ or H_2_S gas injection may be ineffective because the slow oxidation of the gas and preferential condensation on pre-existing particles lead to particles substantially larger than optimal, Pierce et al.^10^ investigated the formation of particles in an aircraft plume with H_2_SO_4_ injection, and calculated nucleation rates by scaling the kinetic barrierless nucleation theory of Clement and Ford^17^ with four scaling factors ranging from 1 to 10^–9^ (to assess the effect of uncertainty in nucleation rate calculation). They showed that, after 2 days of evolution, the particle size distributions in the plume are mostly determined by the H_2_SO_4_ injection and plume dilution rates and are insensitive to nucleation and condensation rate uncertainties, consistent with the analysis of Turco and Yu^14^ with regard to the particle self-coagulation limitation in diluting plumes. Pierce et al.^10^ showed that the introduction of H_2_SO_4_ vapor can allow better control of the particle size distribution, potentially increasing radiative forcing per sulfur mass relative to the introduction of SO_2_ gas. It should be noted that English et al.,^11^ based on one of the scenarios in their global simulations that injected H_2_SO_4_ is instantly well-mixed throughout the grid box (i.e., no plume scale nucleation), showed that such an H_2_SO_4_ injection did not impact SAI efficacy compared to SO_2_ injection. However, when they injected H_2_SO_4_ as sulfate particles, their simulated SAI efficacy was larger than that of the SO_2_ SAI, similar to those of other studies.^8,10^ Regardless, English et al.^11^ suggested that more research on the plume scale processes is needed. Benduhn et al.^13^ explored SAI “steerability” by examining the two key parameters governing self-limited aerosol growth: plume dilution rate (or diffusivity) and initial H_2_SO_4_ concentration. Benduhn et al.^13^ carried out simulations with an aerosol microphysics model linked to H_2_SO_4_–H_2_O binary homogeneous nucleation (BHN) parametrization of Vehkamäki et al.,^18^ and pointed out that properties of aerosols controlled by self-limited aerosol growth do not depend on the actual nucleation rate. Benduhn et al.^13^ showed that the regime satisfying all criteria for controlled generation of desired particles is characterized by a relatively narrow parameter space (i.e., ranges of initial H_2_SO_4_ concentration and plume diffusivity) as well as steep gradients of the sizes of generated particles with regard to initial H_2_SO_4_ concentration that might translate into technical difficulties of implementation. Regarding technical difficulties of implementation, we would like to note that Gao et al.^19^ offered a delivery method using solar energy to loft SAI material injected at lower altitudes (accessible by conventional aircraft) into the stratosphere but pointed out that process-level understanding of subgrid processes is still required.

The above-mentioned plume-scale microphysics studies, while revealing the general importance of self-coagulation in controlling particle properties and better steerability with H_2_SO_4_ injection, have some limitations. First, the mechanisms of particle formation in the H_2_SO_4_ plume remain unclear. The validity of nucleation schemes^17,18^ used in Pierce et al.^10^ and Benduhn et al.^13^ was not robustly interrogated. For example, relevant H_2_SO_4_ concentrations in the plume with H_2_SO_4_ injection are well beyond the H_2_SO_4_ concentration valid range of the Vehkamäki et al.’s parametrization (10^4^–10^11^ cm^–3^).^18^ While both studies pointed out the insensitivities of results to uncertainties in nucleation rate calculations under the limited conditions assumed in these studies, a solid understanding of nucleation processes and controlling parameters is necessary to predict confidently the sizes and concentrations of particles produced under various relevant conditions. Second, the microphysical simulations of Pierce et al.^10^ and Benduhn et al.^13^ did not consider the role of chemiions (i.e., ions generated through chemiionization reactions during fuel combustion) which is important for particle formation in aircraft plumes. As mentioned earlier, NASEM recommends the role of chemiions to be understood.^2^ Third, the concentrations of soot and particles due to impurity (i.e., injected H_2_SO_4_ solution is not 100% pure and contains residuals that do not evaporate) or incomplete evaporation in the initial exhaust can be large enough to scavenge injected H_2_SO_4_ and have not yet been studied. It should be noted that the parametrization of the particle size distribution in an expanding plume given in Turco and Yu^14^ was derived under the assumption that particles are in similar sizes (i.e., one mode) and it is unclear if this parametrization remains valid in a particle system with multiple modes. Finally, the timing (plume age) and conditions under which most particles are formed are unclear. Pierce et al.^10^ initialized their plume aerosol microphysics model with H_2_SO_4_ vapor and background aerosols at *T* = 220 K and RH= 10%. However, it takes some time for the initial hot aircraft exhaust (*T* = ∼ 600 K) to approach ambient *T* (∼220 K) via dilution, during which RH changes rapidly. On the other hand, at *T* = 220 K, the plume is already significantly diluted (by a factor of 100 or more) and H_2_SO_4_ concentrations in the plume should be much smaller than those in the initial plume (i.e., plume age = 0 s). Therefore, the conditions for the nucleation in real exhaust plumes are likely quite different from those assumed by Pierce et al.^10^ Benduhn et al.^13^ did not specify under what conditions (*T* and RH) the nucleation rate was calculated.

In this study, we seek to study nucleation pathways and particle size distribution evolutions in stratospheric aircraft exhaust plumes with H_2_SO_4_ enhancement using a state-of-the-art kinetic H_2_SO_4_–H_2_O ion-mediated and homogeneous nucleation model described in Section 2.1 that addresses the limitations in previous plume-scale microphysics studies mentioned above. Injection altitude and ambient conditions, aircraft information, and plume dilution parametrization are given in Section 2.2. Section 3 presents the results, and Section 4 is a Summary and Discussion.

## 2 Materials and Methods

### 2.1 Plume Kinetic Nucleation and Particle Microphysics Model

The model employed for this study is a parcel model of jet plume aerosol microphysics developed back in the 1990s, with microphysics algorithms and nucleation thermodynamics that have been subsequently improved.^15,20−22^ The key improvements include explicit treatment of the evaporation of neutral and charged clusters, new algorithms to consider ion-dipole interactions that are important for both stability of charged clusters and growth enhancement, development of quasi-unary nucleation scheme for H_2_SO_4_–H_2_O binary nucleation, and extensive usage of thermodynamic data from laboratory measurements and quantum chemistry calculations to constrain the composition and stability of prenucleation clusters.^21−25^ All of these are important for resolving explicitly the dynamic evolution of clusters/particles under extreme conditions such as H_2_SO_4_ SAI in stratospheric plumes, as demonstrated in this study.

The kinetic model explicitly solves the complex interactions among ions, neutral and charged clusters of various sizes, vapor molecules, and pre-existing particles. It was originally developed to overcome several limitations of the classical nucleation model in applications to aircraft wakes.^15,20^ First, the steady-state Boltzmann cluster distribution assumption implied in the classical nucleation model is only approximately valid in rapidly changing aircraft exhaust plumes, where cluster formation and concentrations are limited by kinetics.^15,20^ Second, the bulk capillarity assumption is not strictly valid in a rapidly cooling aircraft plume where the H_2_SO_4_ supersaturations are so high that critical nucleation embryos consist of only a few molecules.^21^ This assumption leads to extremely large uncertainty in the calculated nucleation rates (many orders of magnitude).^20^ Third, the classical nucleation model does not take into account the scavenging of prenucleation clusters by large concentrations of soot particles in the fresh aircraft exhaust. Finally, the classical nucleation model does not have the capability of treating the evolution of chemiions and their influences on cluster formation and particle growth. The kinetic nucleation model developed by Yu and Turco^15^ not only addresses these limitations but also enables continuous improvement of the model by incorporating into it molecular bonding and clustering thermodynamic data derived from experimental measurements and quantum chemistry calculations,^26−33^ which significantly reduces the uncertainty in the predicted particle formation rates and improves agreement with measurements.^20−22^ It should be noted that the improvement and application of the kinetic nucleation model in the last two decades are mostly for the conditions in the background ambient atmosphere, as opposed to those in the rapidly evolving aircraft exhaust plumes.^22^ In the present work, the improved kinetic nucleation model is adapted and implemented back to the original jet plume parcel model for simulating particle formation in the aircraft plume. A similar model was previously applied to study particle formation in anthropogenic SO_2_ plumes.^34^

The kinetic nucleation and aerosol microphysics model uses a discrete-sectional bin structure to represent the sizes of clusters/particles, starting from a single unhydrated H_2_SO_4_ molecule (effective dry diameter 0.54 nm) to particles of tens of micrometers. In this work, we use 112 bins to cover the particle size (diameter) range of 0.54 nm −41.6 μm, with the first 20 bins as discrete bins (i.e., the *i*th bin contains *i* H_2_SO_4_ molecules, *i* ≤ 20).^21^ Three types of aerosols are treated in the model: nucleated sulfuric acid particles, soot/impurity particles, and background aerosols. In the presence of chemiions, sulfuric acid particles are further separated into neutral, positively charged, and negatively charged clusters/particles. The amount of injected sulfuric acid scavenged by soot/impurity particles and background particles through H_2_SO_4_ direct condensation and coagulation of formed sulfuric acid particles by these particles is tracked separately.

### 2.2 Injection Altitude and Ambient Conditions, Aircraft Information, and Plume Dilution

The present study focuses on the aircraft injection of H_2_SO_4_ at an altitude of 20 km which has been simulated by many global model studies.^8^ Typical ambient conditions corresponding to this altitude in the tropics are assumed: pressure = 55 mb, *T* = 217 K, RH = 0.6%, and ionization rate = 12.5 ion-pairs cm^–3^ s^–1^.^35^ The background (relative to freshly injected plume) aerosol is assumed to have a log-normal size distribution with a median diameter of 500 nm and a geometric standard deviation of 1.6, and the total surface area of ambient background aerosol (*S*_background_) is assumed to be 10 μm^2^/cm^3^, corresponding to a potential stratosphere with an enhanced stratospheric aerosol layer due to ongoing SAI.^8^

The exact platforms to be used for the potential delivery of species into the stratosphere remain to be explored or designed.^36^ Rasch et al.’s analysis of plume aerosol microphysics was for a fighter-jet-sized aircraft.^4^ Note that in two previous aerosol microphysics modeling studies on H_2_SO_4_ injection,^10,13^ platform characterization is not specific. In this study of detailed aerosol microphysics in aircraft plume with H_2_SO_4_ injection, we use the characterization of the DLR ATTAS research aircraft used in a previous field campaign: fuel flow rate = 0.164 kg s^–1^, true airspeed = 163 m s^–1^, exit exhaust temperature = 624 K, and jet fuel combustion water emission index = 1.225 kg water kg fuel^–1^, fuel sulfur content = 500 ppm with S-to-H_2_SO_4_ conversion efficiency of 2%, and exit chemiion (positive + negative) concentration = 2 × 10^9^ /cm^3^.^37−39^ The soot particles generated during engine combustion are assumed to have a log-normal size distribution with a median diameter of 45 nm, a geometric standard deviation of 1.6, and an emission index of 10^15^ kg fuel^–1^, which are based on ground-based characterization of soot particle emissions from the DLR ATTAS research aircraft.^37^

For the H_2_SO_4_ injection scheme, it is expected that H_2_SO_4_ will come from liquid sulfuric acid atomized into droplets and then evaporated shortly after injection (into the exhaust manifold). In such a case, residual particles may exit as a result of impurity (not evaporable under the conditions) of liquid sulfuric acid and/or incomplete evaporation of atomized droplets. To account for such a potential effect, we consider another mode of particles (named impurity particles) in the initial particles in the exhaust. The concentrations and size distribution of impurity particles can be calculated from the impurity fraction (*f*_impurity_), sulfur injection rate (SIR), and size distributions of atomized droplets. Due to the lack of information available, the atomized droplets are assumed to have a number median diameter of 10 μm and a geometric standard deviation of 1.6 in the present study. *f*_impurity_ is assumed to be 0.1% for the baseline case, but a sensitivity study is carried out. Two SIR values (0.1 and 3 kg S km^–1^) are compared in detail and a sensitivity study covers SIR ranging from 0.001 to 10 kg S km^–1^.

The dilution or mixing of the aircraft plume is a key process that determines the conditions under which particles (including contrails) form, evolve, and interact.^20^ Schumann et al.^40^ analyzed aircraft exhaust dilution from measurements in more than 70 plume encounters in the upper troposphere and lower stratosphere for plume ages of milliseconds to 95 min and found that the bulk dilution ratio (DR) measured in these encounters under a wide range of conditions can be approximated by
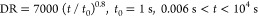
1where *t* is the plume age (in s) and DR is defined as the ratio of the mass of plume gases to the mass of fuel burned per unit flight distance (*m*_fuel_, kg fuel km^–1^) from which the plume cross-sectional area (*A*) can be calculated as
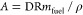
2where ρ is the air density within the exhaust plume.

In this study, our plume simulation starts at *t* = 0.003 s and we use Equation 1 to calculate DR for *t* < 10^4^ s. For *t* ≥ 10^4^ s, we use the following equation to parametrize the dispersion of the plume in the stratosphere^41^
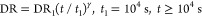
3where DR_1_ is the dilution ratio at *t* = t_1_ from eq 1 and γ is the dilution exponent coefficient that depends on atmospheric stability and wind shear (γ = 1.5 is used in this study).

It should be noted that our simplified dilution parametrization assumes uniform mixing across the plume cross-section, representing “average” conditions within the exhaust plume. With a given SIR, H_2_SO_4_ vapor concentration (C_H2SO4_, in # molecules/cm^3^) at the engine exit is calculated as
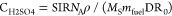
4where *N*_A_ is the Avogadro’s number and *M*_S_ is the molecular weight of sulfur. DR_0_ is the bulk dilution ratio at the engine’s exit point.

## 3 Results

**Figure 1:**
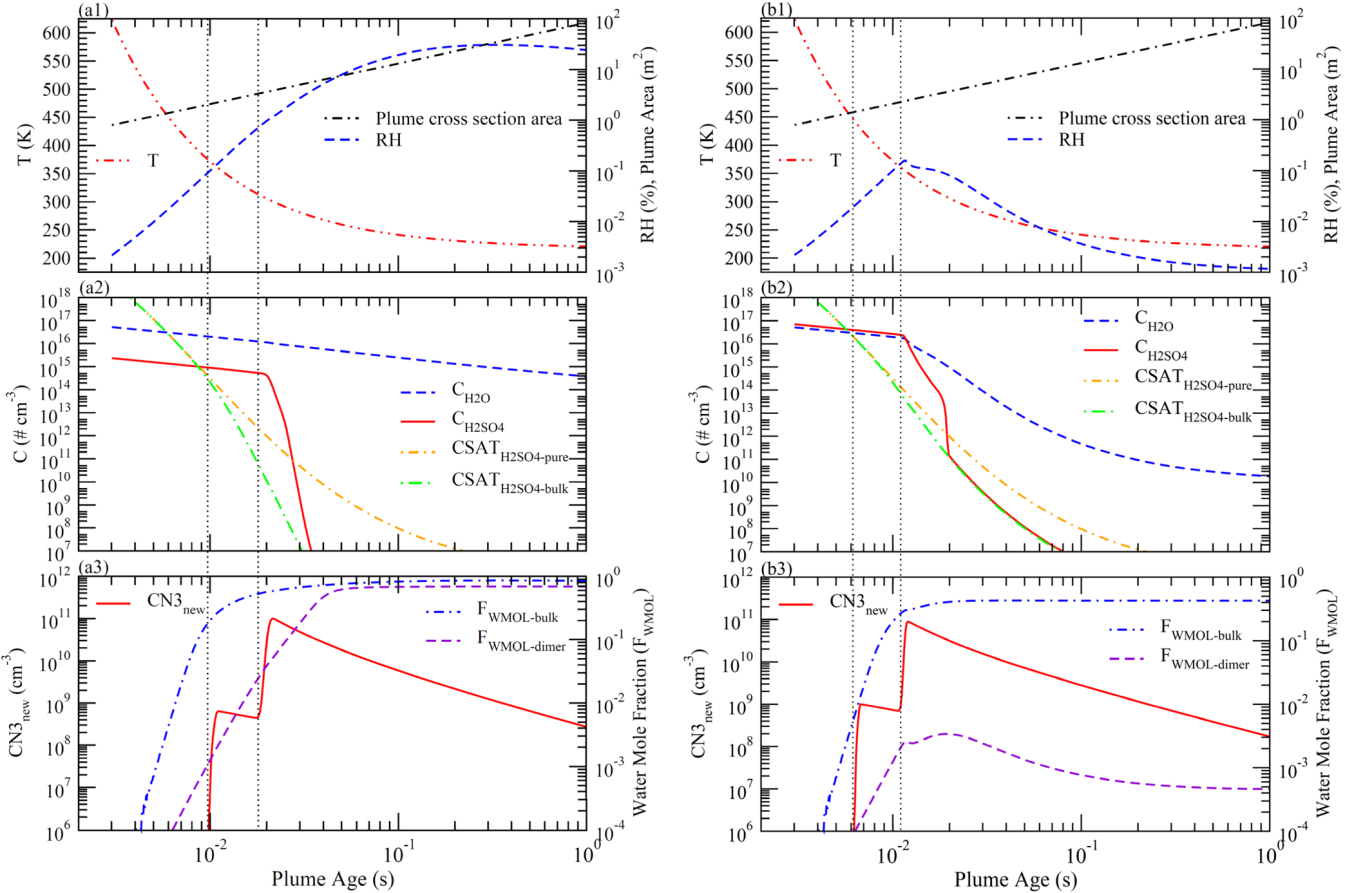
(a) Time evolution of selected variables in exhaust plumes shortly after emissions (plume age 0.003–1 s) with H_2_SO_4_ injection rates of 0.1 kg S km^–1^. (1) temperature (*T*), relative humidity (RH), and plume cross-sectional area; (2) concentrations of water vapor (C_H2O_) and sulfuric acid vapor (C_H2SO4_), saturation H_2_SO_4_ concentrations over pure H_2_SO_4_ solution (CSAT_H2SO4-pure_) and over bulk H_2_SO_4_–H_2_O solution in equilibrium with water vapor (CSAT_H2SO4-bulk_); and (3) condensation nuclei with a dry diameter larger than 3 nm (CN3), mole fraction of water molecules in bulk solution (F_WMOL-bulk_) and sulfuric acid dimers (F_WMOL-dimer_) in equilibrium with water vapor. Two vertical dotted lines show the plume ages when ion nucleation (left) and neutral nucleation (right) start. (b) Same as Figure 1a except with H_2_SO_4_ injection rates of 3 kg S km^–1^.

**Figure 2:**
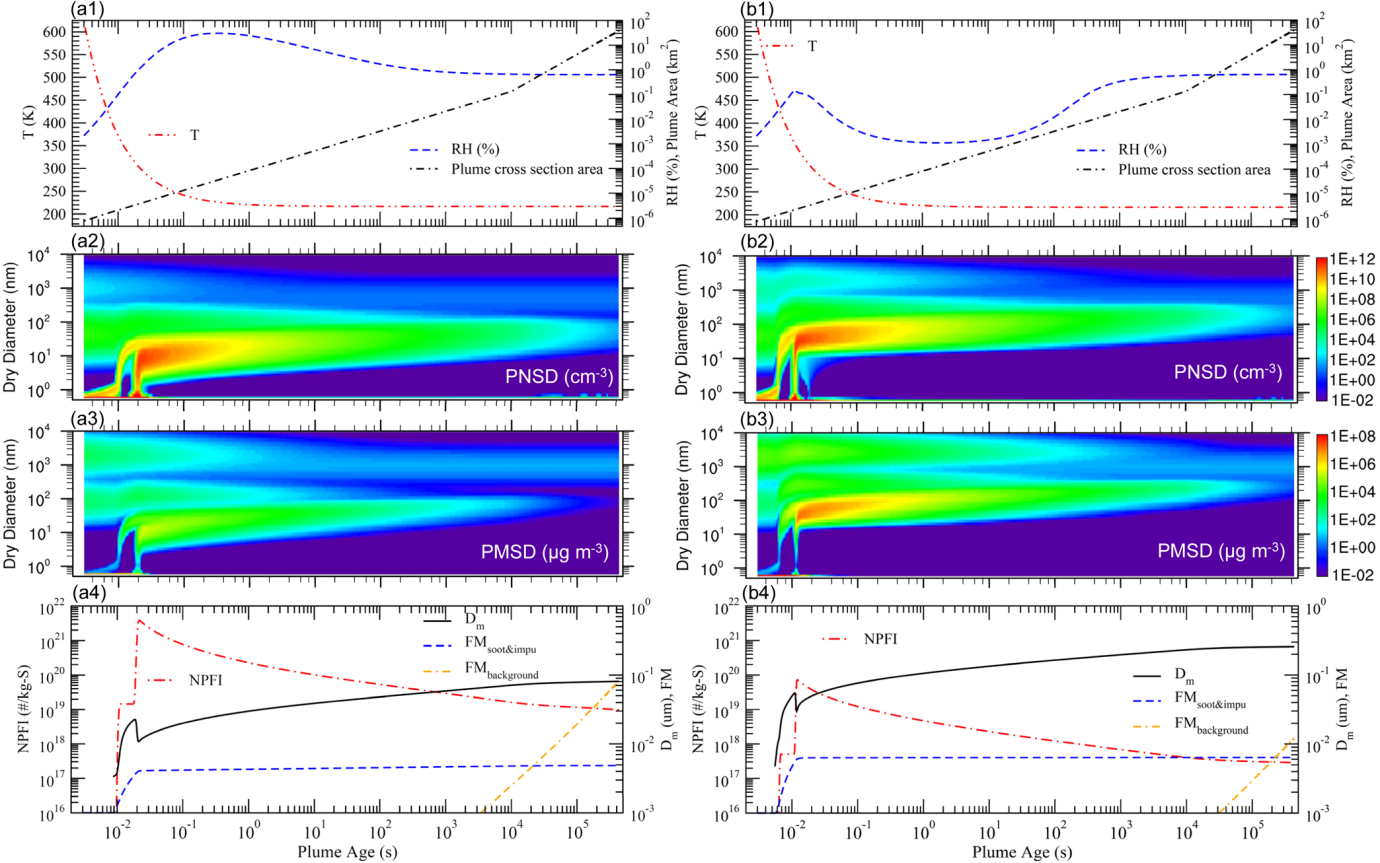
Five-day time evolution of selected variables and particle size distributions in exhaust plumes with H_2_SO_4_ injection rates of 0.1 kg S km^–1^ (a) and 3 kg S km^–1^ (b). (1) temperature (*T*), relative humidity (RH), and plume cross-sectional area; (2) particle number size distribution (PNSD) d*N*/d*l*ogDp; (3) particle mass size distribution (PMSD) d*M*/d*l*ogDp; and (4) formation index of new particles (CN3) per kg S injected (NPFI), mass-weighted mean diameter of new particles (>3 nm) (Dm), fraction of injected sulfur mass ended up in soot and impurity particles (FM_soot&impu_) and in background particles (FM_background_).

Using the model described in Section 2, we carried out simulations of detailed particle microphysics in the exhaust plume from the exit point to a plume age of 5 days under two H_2_SO_4_ injection rates (SIR = 0.1 and 3 kg S km^–1^). Figures 1 and 2 present the evolution of plume thermodynamics, key variables of our interest, and particle size distributions, with Figure 2 showing the whole 5-day period and Figure 1 zooming into the first second for selected variables.

### 3.1 Plume Thermodynamics and RH

In Figures 1a1,b1 and 2a1,b1, the growth in the cross-sectional area of the plume is calculated with the parametrizations described in Section 2, and the plume *T* is calculated using the corresponding dilution ratio. The plume *T* drops rapidly after emission as a result of mixing with cold ambient air, approaching the ambient level at a plume age of ∼1 s when the initial exit exhaust has already been diluted by a factor of around 100. The decrease in *T* leads to an initial increase of RH, reaching a maximum of 30% at a plume age of 0.3 s for the case with SIR = 0.1 kg S km^–1^ (Figures 1a1 and 2a1) but the change of RH for the case with SIR = 3 kg S km^–1^ is much more complex due to uptake of water to the H_2_SO_4_ particles, which will be discussed next. For both cases, plume RH approaches the ambient level (0.6% assumed in this study) at plume ages of around 1000 s (Figure 2a1,b1).

In most situations in the atmosphere where H_2_SO_4_–H_2_O nucleation occurs, the concentration of H_2_O vapor (C_H2O_) is much larger than that of H_2_SO_4_ (C_H2SO4_), and the fraction of H_2_O taken up by newly formed sulfuric acid particles is negligible. While this is the case in the plume with a low SIR of 0.1 kg S km^–1^ (Figure 1a2), it is no longer true in the plume with an SIR of 3 kg S km^–1^ where C_H2SO4_ is slightly higher than C_H2O_ in the initial exhaust plume (Figure 1b2). Under the configuration of the platform specified in this study (Section 2), to achieve newly formed particles with diameters in the range of 200–300 nm (Figure 2), a H_2_SO_4_ injection rate at a magnitude of ∼3 kg S km^–1^ is needed. Therefore, under this injection scenario, the effect of the consumption of water vapor by formed particles on RH and thus particle microphysics needs to be considered. Our plume microphysics model takes into account this effect by explicitly solving the partitioning of water vapor (from both fuel combustion production and ambient air mixed in) in gas and particle phases. As shown in Figures 1b1 and 2b1, RH in the exhaust plume with SIR of 3 kg S km^–1^ increases initially (up to plume age of ∼0.012 s) due to quick cooling but decreases thereafter due to consumption of water vapor by newly formed sulfuric acid particles, approaching a minimum of ∼0.001% at plume age of 1.8 s. As the plume continues to evolve (after 1.8 s), RH gradually increases due to the ambient water vapor mixed in, ultimately approaching the ambient RH of 0.6% at a plume age of around 1000 s. At a plume age of 0.2 s, RH in the plume with SIR = 3 kg S km^–1^ is 4 orders of magnitude lower than that with SIR = 0.1 kg S km^–1^, highlighting the critical importance of explicitly solving the partitioning of water between the vapor and aerosol phase for SAI at high SIR rates.

### 3.2 Nucleation Processes: Ion Mediated versus Neutral Homogeneous Nucleation

With the direct H_2_SO_4_ injection, it is not surprising that C_H2SO4_ in the initial plume is high, reaching 2.36 × 10^15^ and 7.09 × 10^16^ cm^–3^ at SIR of 0.1 and 3 kg S km^–1^, respectively. What is surprising is that H_2_SO_4_ is supersaturated in the plume at a very young age (*t* < 0.02 s) and at a quite high temperature (*T* in the range of 310–445 K), not only over the bulk binary sulfuric acid solution but also over pure liquid sulfuric acid (Figure 1a2,b2).

As can be seen from Figure 1a3,b3 (also Figure 2), the formation of new particles occurs rapidly at plume ages of 0.01–0.02 s for SIR = 0.1 kg S km^–1^ and 0.006–0.012 s for SIR = 3 kg S km^–1^ (Figure 1a3,b3). Compared to neutral homogeneous nucleation, nucleation on ions (or chemiions generated during combustion) has advantages due to enhanced stability and growth rate of charged clusters.^15,20−22^ As a result, nucleation on chemiions occurs at younger plume age and higher *T*: at *t* = 0.01 s, *T* = 375 K for SIR of 0.1 kg S km^–1^ and *t* = 0.006 s, *T* = 445 K for SIR of 3 kg S km^–1^. In contrast, neutral homogeneous nucleation occurs at *t* = 0.02 s, *T* = 310 K for SIR of 0.1 kg S km^–1^ and *t* = 0.012 s, *T* = 360 K for SIR of 3 kg S km^–1^. While particles formed on chemiions grow faster and larger (Figure 2a2–a4,b2–b4), their concentrations are limited by chemiion concentrations and the amount of injected H_2_SO_4_ mass consumed by these particles are relatively small, which can be seen from no obvious change in the trend of C_H2SO4_ after the onset of ion nucleation (Figure 1a2,b2). It is only after the onset of homogeneous nucleation that C_H2SO4_ drops rapidly and CN3 increases to a maximum of around 10^11^ cm^–3^ for both SIR cases. Thereafter, nucleated particles grow mainly via self-coagulation.

### 3.3 Nucleation Processes: H_2_SO_4_ Unary versus H_2_SO_4_–H_2_O Binary Nucleation

As mentioned earlier, in the plume with H_2_SO_4_ injection, C_H2SO4_ is supersaturated over pure liquid shortly after emission when the temperature is still quite high (∼310–445 K) and RH is very low (< ∼0.1%) (comparing the red solid line with the orange dot-dashed line in Figure 1a2,b2). Under such conditions, new particles can form via unary pure H_2_SO_4_ nucleation (i.e., without the participation of H_2_O), especially in the case with SIR = 3 kg S km^–1^. This can be seen from the mole fraction of water molecules in the bulk solution (F_WMOL-bulk_) and H_2_SO_4_ dimers (F_WMOL-dimer_) in equilibrium with water vapor (Figure 1a3,b3). For the case with SIR = 0.1 kg S km^–1^, F_WMOL-dimer_ is 7.9 × 10^–4^ and 0.027 while F_WMOL-bulk_ is 0.14 and 0.54 upon the onset of ion nucleation and neutral nucleation, respectively. For the case with SIR = 3 kg S km^–1^, F_WMOL-dimer_ is 6.6 × 10^–5^ and 0.002 while F_WMOL-bulk_ is 3.3 × 10^–3^ and 0.27 upon the onset of ion nucleation and neutral nucleation, respectively. For small clusters and particles, F_WMOL_ depends on particle sizes because of the Kelvin effect and approaches the bulk value as the particle sizes increase, which is considered in our kinetic nucleation model.^21^ Based on the temporal evolution of F_WMOL-dimer_ and F_WMOL-bulk_ as shown in Figure 1a3,b3, it is clear that ion nucleation proceeds via unary H_2_SO_4_ nucleation (i.e., the fraction of water in initially formed clusters/particles is negligible) for both SIR = 0.1 and 3 kg S km^–1^. For neutral nucleation, it is primarily unary for SIR = 3 kg S km^–1^ but is binary for SIR = 0.1 kg S km^–1^. It should be noted that previous studies of SAI with H_2_SO_4_ injection assume either H_2_SO_4_–H_2_O binary nucleation or kinetic barrierless nucleation of H_2_SO_4_ with scaling factors ranging from 1 to 10^–9^.^10,13^ This work shows that the nucleation in the plumes with H_2_SO_4_ injection can be dominated by either a binary or unary process, depending on SIR values.

### 3.4 Aging of Nucleated Particles in the Plume and Scavenging by Pre-Existing Particles

For SAI, it is important to understand the aging of newly formed particles in the subgrid plume as the injected plume dilutes to the size of a typical global model grid box or to ambient level concentrations. Figure 2 presents a 5-day evolution of plume thermodynamics and particle properties for two scenarios with SIR = 0.1 and 3.0 kg S km^–1^. For the small research airplane platform and dilution process considered in this study (Section 2), the plume cross-sectional area reaches 37.3 km^2^ by the plume age of 5 days. After the initial quick formation of particles within ∼0.02 s of plume age, the evolution of particle size distributions in the plume is dominated by coagulation (and mixing with ambient air). A key concern of SAI is the amount of injected mass ending up growing pre-existing particles, especially those relatively larger particles already in the stratosphere, rather than generating new particles. To investigate this, our aerosol model treats newly formed particles, soot and impurity particles, and ambient particles separately.

In the initial plume (*t* = 0.003 s), only soot and impurity particles exist (Figure 2a2,a3,b2,b3), noting that the absolute mass (and number) concentrations of impurity particles depend on SIR and *f*_impurity_, and *f*_impurity_ can also be used to account for potential incomplete evaporation of injected H_2_SO_4_ droplets (Section 2). Under the assumed sizes of initial atomized sulfuric acid droplets and f_impurity_ (Section 2), the impurity particles have a number median size of 1 μm and total number concentrations of 1.52 × 10^2^ # cm^–3^ for SIR = 0.1 kg S km^–1^ and 4.55 × 10^3^ # cm^–3^ for SIR = 3.0 kg S km^–1^ As the plume evolves, new particles form during a very short period of time while ambient particles mix in continuously. Around the time of ion nucleation, when H_2_SO_4_ is supersaturated, condensation of H_2_SO_4_ on pre-existing particles also occurs. From PNSD and PMSD evolution plots, the growth of soot and impurity particles can be clearly seen, especially for the case of SIR = 3 kg S km^–1^, where the sizes of soot particles more than doubled due to high C_H2SO4_. The condensation of H_2_SO_4_ grows the particles nucleated on ions quickly to reach a mass-weighted mean diameter (*D*_m_) of 22.7 nm for SIR = 0.1 kg S km^–1^ and 54.9 nm for SIR = 3.0 kg S km^–1^ by the time neutral nucleation starts. Neutral nucleation increases particle number concentrations but decreases overall *D*_m_ (see PNSD and PMSD as well as solid lines in Figure 2a4,b4). After the completion of neutral nucleation, *D*_m_ increases gradually via coagulation and reaches 80.5 nm with an SIR of 0.1 kg S km^–1^ and 256.6 nm with an SIR of 3.0 kg S km^–1^ at a plume age of 5 days.

Coagulation increases particle sizes but decreases particle number concentrations, which can be seen from the evolution of particle size distributions as well as D_m_ and new particle formation index (NPFI) (red dot-dashed lines in Figure 2a4,b4). NPFI is normalized to SIR to eliminate the variation in the number concentrations due to plume dilution effects and is in the unit of number of particles formed per kg of injected S. NPFI has a value of 9.74 × 10^18^ kg S^–1^ for SIR of 0.1 kg S km^–1^ and 2.89 × 10^17^ kg S^–1^ for SIR of 3.0 kg S km^–1^, roughly inversely proportional to SIR. This inverse dependence of NPFI on SIR is a result of aerosol number concentration invariance in a coagulating and diluting plume^14^ and the definition of NPFI (=CN3/(DR*SIR)). The self-coagulation leads to a narrow size distribution of the newly formed particles at the plume age of 5 days, with a geometric standard deviation of ∼1.3 which is close to the asymptotic geometric standard deviation of log-normally preserving size distribution for Brownian coagulation.^42^

Figure 2a4, b4 also show the fraction of injected S mass (FM) scavenged by soot/impurity particles (dashed blue lines, FM_soot&impu_) and ambient background particles (dot-dashed orange lines, FM_background_). It can be seen that most scavenging by soot and impurity particles is due to condensation before the onset of neutral nucleation. Thereafter, the scavenging slows as a result of (1) fast dilution of soot/impurity particles and (2) an increase in the sizes of nucleated particles, which reduces coagulation scavenging coefficients of newly formed particles by soot/impurity particles. It should be noted that FM_soot&impu_ depends on initial concentrations (and sizes) of soot and impurity particles in the exhaust. FM_soot&impu_ at *t* = 5 days for 3.0 kg S km^–1^ is 0.0064 (or 0.64%) which is higher than that for 0.1 kg S km^–1^ case (0.0049 or 0.49%), as a result of more absolute concentrations of impurity particles (see Figure 2a2,a3,b2,b3). These values are based on *f*_impurity_ = 0.1%. Currently, there is little information about the possible values of *f*_impurity_ (note that we treat incomplete evaporation as a part of *f*_impurity_), and a sensitivity analysis is given later.

The scavenging of injected sulfur by background particles becomes important after the plume is well mixed with ambient air, at plume age *t* > ∼10^3^ s for SIR = 0.1 kg S km^–1^ and *t* > ∼ 10^4^ s for SIR = 3.0 kg S km^–1^. With fixed concentrations (and sizes) of background aerosols, coagulation scavenging coefficients and hence FM_background_ depend on the size difference between nucleated and background particles. Under the size distribution of background particles assumed in this study, FM_background_ at a plume age of 5 days is 0.012 (or 1.2%) for SIR = 3.0 kg S km^–1^ and is a factor of 6.5 larger for SIR = 0.1 kg S km^–1^ (0.078, or 7.8%). Since the size of nucleated particles for SIR = 3.0 kg S km^–1^ is around the optimal size for scattering efficiency per mass, the smaller FM_background_ confirms the benefit of the proposed H_2_SO_4_ injection in achieving the desired sizes and reducing loss of injected sulfur to pre-existing background particles.^10,16^

### 3.5 Sensitivity Studies To Understand the Impacts of Key Parameters

In the case studies shown in Figures 1 and 2, we assume representative values for key parameters that are subject to large variations or uncertainties. Here we explore the impacts of some key parameters through sensitivity studies. One key parameter influencing particle formation and evolution in aircraft exhaust plumes is the dilution ratio. In our baseline simulation, the average dilution (AD) parametrization (eq 1) derived from various measurements by Schumann et al.^40^ is used. In the Supporting Information (Figure S1), we derive two additional fitting parametrizations from more than 70 aircraft exhaust dilution measurements compiled by Schumann et al.,^40^ one roughly representing slow dilution (SD) while the other fast dilution (FD) as given below:
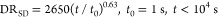
5
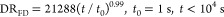
6

**Figure 3:**
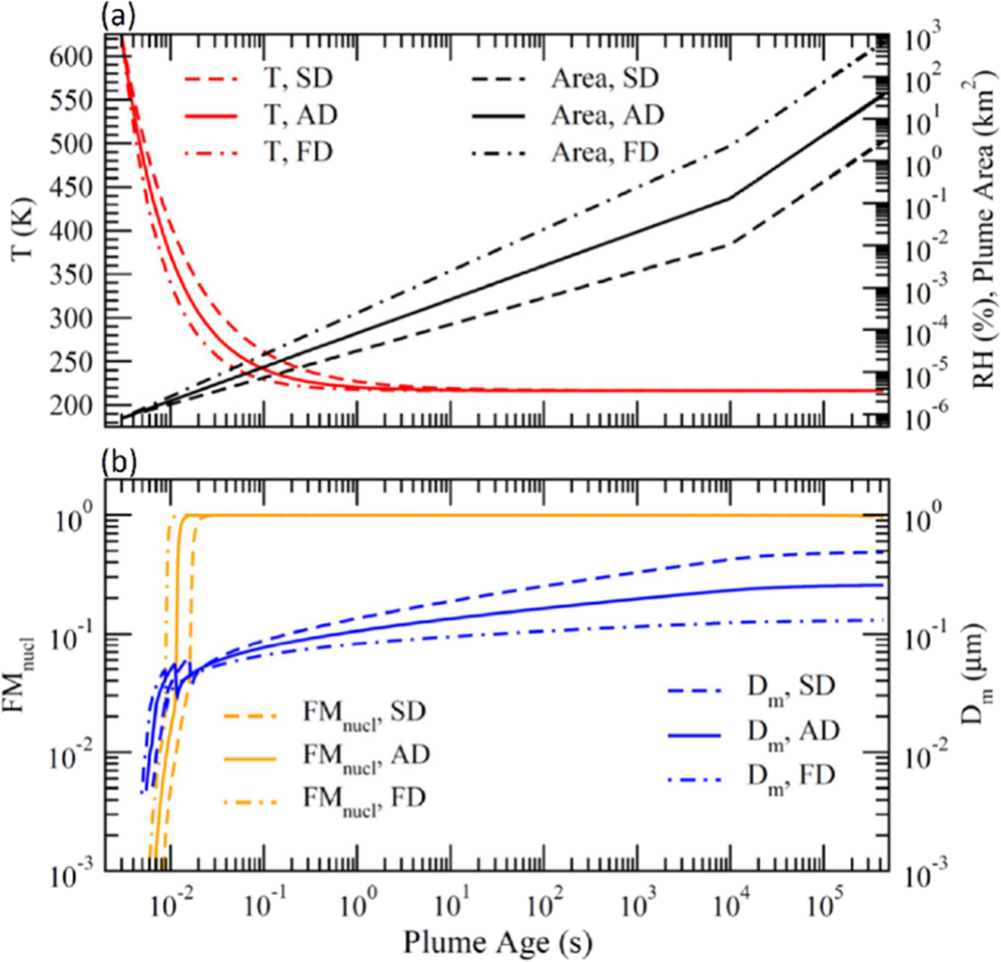
Five-day time evolution of selected variables in exhaust plumes with H_2_SO_4_ injection rates of 3 kg S km^–1^ under three dilution conditions representing slow (SD), average (AD), and fast (FD) dilution. (a) temperature (*T*) and plume cross-sectional area; (b) fraction of injected sulfur mass ended up in nucleated particles (FM_nucl_) and *D*_m_.

Figure 3 shows the evolution of plume *T*, plume cross-sectional area, fraction of injected sulfur mass ended up in nucleated particles (FM_nucl_), and (*D*_m_) in exhaust plumes with H_2_SO_4_ SIR of 3 kg S km^–1^ under three dilution conditions representing slow (SD), average (AD), and fast (FD) dilution, with other parameters the same as those in the baseline case. As expected, slower dilution results in slower expansion of the plume cross-sectional area and decrease of plume *T* (Figure 3a), shifting the nucleation starting time a few milliseconds later (Figure 3b). The dilution ratio has a significant impact on *D*_m_ at the plume age of 5 days, which is 487, 257, and 130 nm for SD, AD, and FD, respectively (Figure 3b). Faster dilution leads to lower concentrations of sulfuric acid vapor and particles formed in the plume, reducing particle growth rates via condensation and coagulation. It should be noted that while the sizes of nucleated particles are smaller for the FD case, the total integrated number of particles formed per kilogram of S injected (i.e., NPFI) is much larger (not shown). In all three dilution cases, most of the injected sulfur ends up in the nucleated particles, with FM_nucl_ = 0.99, 0.98, and 0.96 for SD, AD, and FD, respectively. The slight difference in FM_nucl_ is associated with the difference in the sizes of nucleated particles and thus their scavenging rate by background particles. The high sensitivity of *D*_m_ to dilution ratios highlights the importance of considering the dilution process in the strategy to generate particles of the desired sizes for SAI.

**Figure 4:**
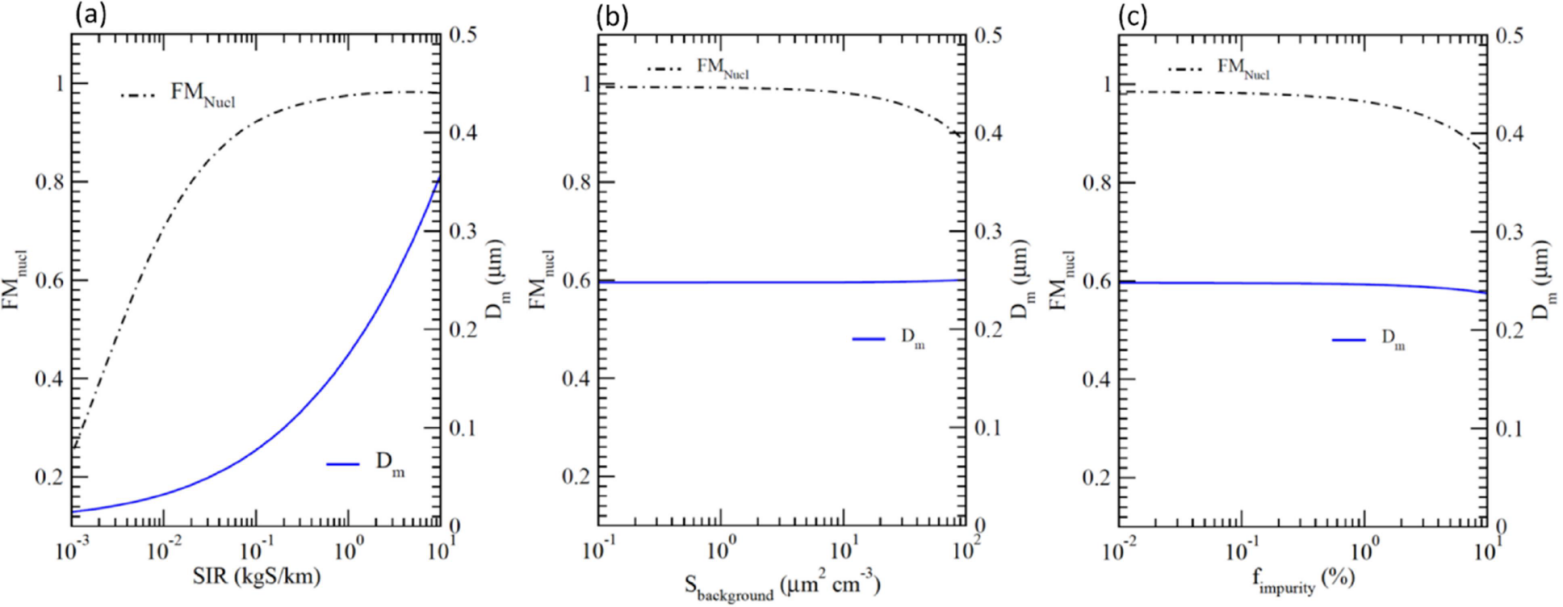
Impacts of (a) SIR, (b) background aerosol surface area (*S*_background_), and (c) impurity fraction (*f*_impurity_, including incomplete evaporation) on FM_nucl_ and *D*_m_ at plume age of 5 days.

Figure 4 illustrates the impacts of SIR, *S*_background_, and *f*_impurity_ on FM_nucl_ and *D*_m_ at the plume age of 5 days. In each sensitivity study, all parameters except the one studied are the same as those in the baseline case. As expected, *D*_m_ increases monotonically with SIR. FM_nucl_ increases rapidly with increasing SIR when SIR < 1 kg S km^–1^ but the increase levels off when SIR > 1 kg S km^–1^. Under the conditions assumed, FM_nucl_ is sensitive to *S*_background_ when *S*_background_ > ∼10 μm^2^/cm^3^ and to *f*_impurity_ when *f*_impurity_ > ∼1% while the effect of both *S*_background_ and *f*_impurity_ on *D*_m_ is quite small. FM_nucl_ at *t* = 5 days are 97.67, 92.39, and 86.6% for *f*_impurity_ = 1, 5, and 10%, respectively. Our simulations indicate that the loss of injected sulfur to soot/impurity particles would be lower with reduced concentrations of soot/impurity particles and reduced nucleation time, which might be achieved by controlling initial exhaust *T* (or H_2_SO_4_ injection location) and ion concentrations and/or dilution processes shortly after emission (*t* < 0.02 s).

In addition to those shown in Figures 3 and 4, our sensitivity studies indicate negligible effects of ambient *T* and RH on FM_nucl_ and *D*_m_. The effect of initial chemiion concentration is also small because of the limit of its concentration by ion–ion recombination and the dominance of neutral nucleation under the conditions examined. However, our simulations did indicate that nucleation on ions occurs first, and chemiions may matter at low SIR (and/or very fast dilution). Future work will more broadly explore the parameter space.

## 4 Summary and Discussion

Solar climate interventions including stratospheric aerosol injection (SAI) have received increasing attention due to climate change risks. NASEM recommended that the US federal government establish an open and internationally collaborative research program to improve SAI knowledge.^2^ One of the research priorities for SAI identified by NASEM is to address critical knowledge gaps in the evolution of the particle size distribution, specifically, to explore plume dynamics, particle nucleation, and subsequent growth, and how implementation choices impact outcomes.^2^ It should be noted that while coagulation is important in governing the evolution and properties of stratospheric aerosols, other processes (particle formation, growth, mixing, and deposition) are likely to be important for SAI efficacy as well. We add that in a realistic SAI scenario, the stratospheric aerosols are unlikely to be in a steady state (or equilibrium) because aerosols (or precursors) to be injected (continuously) are unlikely to be spatially or temporally homogeneous. In such situations, plume scale processes are important.

Here, a state-of-the-art kinetic H_2_SO_4_–H_2_O ion-mediated and homogeneous nucleation model is employed to study the formation of particles in aircraft plumes with H_2_SO_4_ injection. We show that an initial H_2_SO_4_ concentration of ∼7 × 10^16^ cm^–3^ generates particles of optimum sizes of 200–300 nm (for conditions assumed in the present study) and that under such conditions nucleation occurs at very young plume age of 0.006–0.01 s when the plume temperature is quite high (360–445 K) while relative humidity is very low (0.01–0.1%). Nucleation on chemiions preferentially occurs first, followed by neutral nucleation, which converts most of the sulfuric acid vapor to the particle phase in a very short time period (within ∼0.01 s). At the H_2_SO_4_ injection rate to achieve desirable sizes of particles for SAI (SIR = 3.0 kg S km^–1^), the uptake of water vapor by sulfuric acid particles significantly affects water vapor concentration and RH in the fresh plume (*t* < ∼100 s) and nucleation is dominated by H_2_SO_4_ unary rather than binary as H_2_SO_4_ vapor is supersaturated with respect to pure sulfuric acid solution and the water content of initial clusters is close to zero. After the rapid conversion of all injected H_2_SO_4_ vapor to newly formed particles, coagulation (along with dilution) governs the particle size distribution evolution, and the newly formed particles by the plume age of 5 days have a desired narrow size distribution with a geometric standard deviation of ∼1.3. The scavenging of injected H_2_SO_4_ mass is negligible by engine combustion soot particles but can be important by residual particles from injected droplets when the fraction of impurity or incomplete evaporation is substantial. The fraction of injected H_2_SO_4_ mass scavenged by background particles depends on the concentrations of these particles, as well as the sulfur injection rate that affects the sizes of particles formed, and is less than 1% at a plume age of 5 days with ambient particle surface area of 10 μm^2^/cm^3^ and SIR needed for desirable particle sizes.

The present study is subject to the uncertainties associated with the thermodynamics of pure sulfuric acid nucleation at extremely high concentrations of H_2_SO_4_ vapor as well as the dilution process of aircraft exhaust, and sensitivity studies have been carried out to understand the effect of some key parameters on the outcome of H_2_SO_4_ SAI at the plume age of 5 days. The present kinetic model assumes that H_2_SO_4_ clusters and particles of various sizes have average compositions in equilibrium with water. While this assumption is generally valid in the ambient atmosphere, where the concentration of water molecules is much higher than that of H_2_SO_4_ molecules, it may be invalid in the rapidly diluting aircraft exhaust plumes with H_2_SO_4_ enhancement. In addition, various thermodynamic data (including laboratory data, quantum calculation, and capillarity approximation for larger clusters) used in the present model, as detailed in Yu et al.,^22^ are subject to uncertainties. While the prediction of our kinetic model agrees quite well with Cosmics Leaving Outdoor Droplets (CLOUD) measurements^43,44^ under typical ambient atmospheric conditions,^45^ the performance of the model under conditions in aircraft plumes with various levels of H_2_SO_4_ enhancement remains to be evaluated. H_2_SO_4_ gas concentrations considered in CLOUD measurements are much lower than the cases studied here for aircraft exhaust plumes with H_2_SO_4_ enhancement. In addition, the temperature in the plume when nucleation occurs (360–445 K) is also beyond the range of CLOUD measurements (around or below room temperature). In our kinetic nucleation model, a large fraction of thermodynamics data used is changes in enthalpy (Δ*H*) and entropy (Δ*S*) for the formation of prenucleation clusters, which were derived from experimental measurements and quantum chemistry calculation. There are no validity ranges available for these data. Nevertheless, the parametrizations of bulk saturation vapor pressure, surface tension, and density used in the model have validity ranges of 190–298, 230–305, and 230–305 K, respectively.^19,46^ Indeed, the finding of this study indicates that nucleation in H_2_SO_4_-enhanced aircraft plumes occurs at temperatures above these valid ranges. Apparently, real-world experimental data, such as laboratory and field measurements under these high *T* and high H_2_SO_4_ concentrations, can help reduce the model uncertainties and improve our understanding. Another aspect to consider is the potential for contrail formation, which could modify particle formation and evolution in SAI plumes. Although contrails are unlikely to persist at the flight altitudes proposed for SAI, owing to the dry conditions in the stratosphere, short-lived contrail ice formation is possible at sufficiently low temperatures. Such short-lived contrails are improbable for the SIR values that yield the desired particle sizes (diameter ∼200–300 nm), as all water vapor from engine combustion would undergo uptake by the injected sulfuric acid. However, the formation of short-lived contrails is possible at sufficiently low temperatures for H_2_SO_4_ SAI at low injection rates or SO_2_ SAI. The exact conditions favoring contrail formation and implications for SAI require further investigation.

Our sensitivity study shows a significant impact of dilution rates on the sizes of particles formed in the plume. It is unclear how good the dilution parametrization of Schumann et al.^40^ used in this study reflects the conditions or stability at 20 km altitude in the tropics, especially shortly after emissions (*t* < ∼0.01 s) when most of the gas to particle conversion occurs. For example, an SAI-specialized aircraft flying at 20 km altitude is likely to fly with an optimized high-bypass engine that may have different dilution characteristics. The location of H_2_SO_4_ injection may also affect the initial dilution rate and, thus, particle formation and sizes. Future studies using outputs from computational fluid dynamics (CFD) may help us assess these uncertainties and optimize H_2_SO_4_ injection strategies. In addition, the likely inhomogeneity in dilution across the plume cross-section may differentiate the nucleation and growth and thus particle size distributions, and studies using more accurate plume dispersion model alternatives such as the multilayered plume or a fully coupled LES-microphysics simulation should be carried out.^47−49^ It should also be pointed out that the present simulations are limited to plume ages of up to 5 days. Previous work studying volcanic eruptions found that it took several months for aerosol effective radius to reach its maximum.^50,51^ Integration of subgrid plume scale process into global aerosol models is needed for long-term simulations of the efficacy of H_2_SO_4_ SAI. In summary, further modeling studies along with laboratory and in situ measurements are needed to reduce the uncertainty and advance our understanding of nucleation, aerosol dynamics, and plume dispersion so that we can confidently predict the timing and properties of the particle size distribution for a given input of aerosol or its precursor over a range of altitudes and latitudes.^2^

## Supporting Information Available

Dilution ratio as a function of the plume age based on more than 70 measurements (PDF)
