# Supplementary material for: On Nucleation Pathways and Particle Size Distribution Evolutions in Stratospheric Aircraft Exhaust Plumes with H2SO4 Enhancement
Source: Environ Sci Technol. 2024 Apr 9;58(16):6934–44. doi: 10.1021/acs.est.3c08408 (PMC11044588; doi:10.1021/acs.est.3c08408)
Supplement: Supplementary file 1 — es3c08408_si_001.pdf [file es3c08408_si_001.pdf]

Supporting Information (SI)

On nucleation pathways and particle size distribution evolutions in stratospheric aircraft exhaust plumes with H_2SO_4 enhancement

Fangqun Yu[1], Bruce E. Anderson[2], Jeffrey R. Pierce[3], Alex Wong[4], Arshad Nair[1], Gan Luo[1], and Jason Herb[1]*

[1]Atmospheric Sciences Research Center, State University of New York, Albany, New York 12226, United States

[2] Science Directorate, NASA Langley Research Center, Hampton, Virginia 23666, United States

[3] Department of Atmospheric Science, Colorado State University, Fort Collins, Colorado 80521, United States

[4] SilverLining, Washington, D.C. 20001, United States

Number of pages: 2

Number of figures: 1

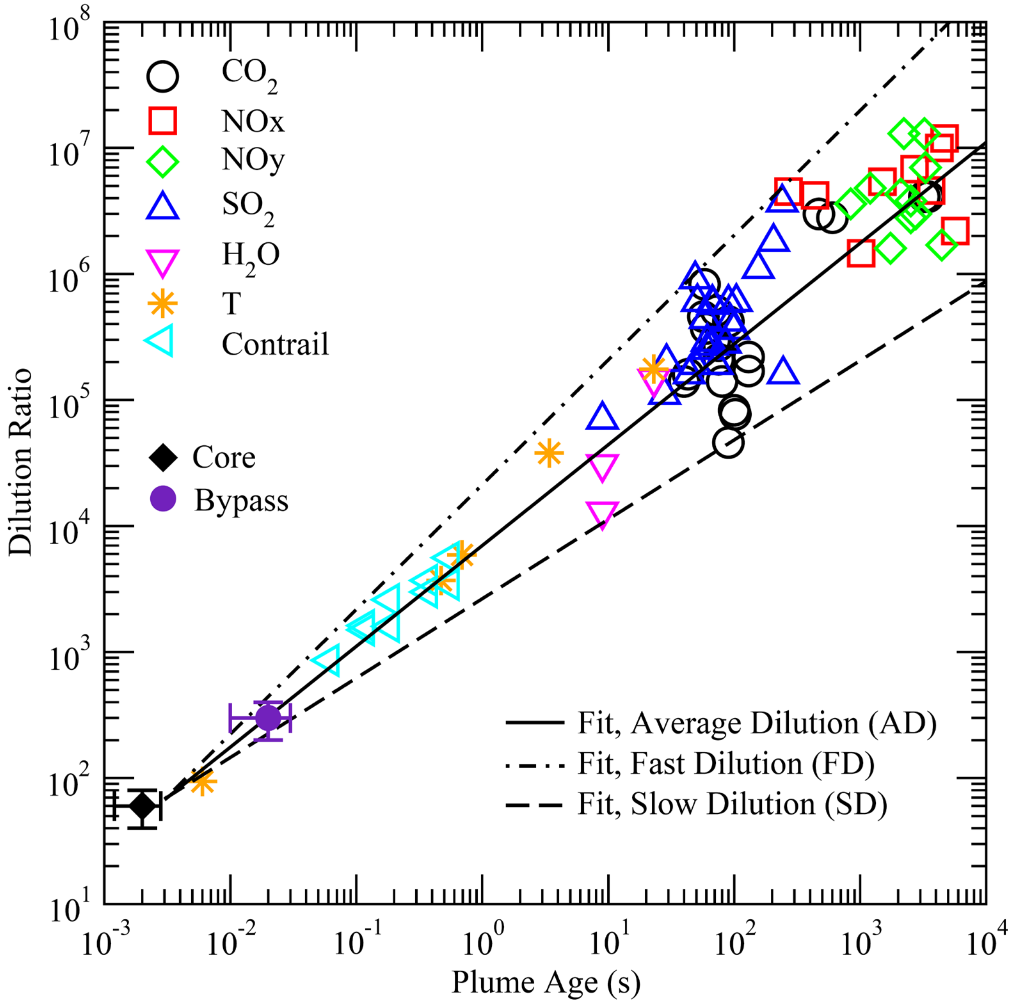

Figure S1. Dilution ratio as a function of plume age based on more than 70 measurements (symbols without error bars) as reported in Schumann et al. (1998). The symbols with error bars denote characteristic values for the engine core and bypass exits (Schumann et al., 1998). The dashed, solid, and dash-dotted lines are fitting curves corresponding to slow dilution (SD), average dilution (AD), and fast dilution (FD) (see Equations 1, 5, & 6 in the main text).

References

Schumann, U., Schlager, H., Arnold, F., Baumann, R., Haschberger, P., and Klemm, O.: Dilution of aircraft exhaust plumes at cruise altitudes. *Atmospheric Environment*, *32*(18), 3097–3103. https://doi.org/10.1016/S1352-2310(97)00455-X, 1998.
